# Automatic system for the determination of metals by anodic stripping potentiometry in non-deaerated samples

**DOI:** 10.1155/S146392469000013X

**Published:** 1990

**Authors:** A. Cladera, J. M. Estela, V. Cerdá

**Affiliations:** Department of Chemistry Faculty of Sciences University of the Balearic Islands Palma de Mallorca E07071 Spain

## Abstract

An automatic system for the determination of Zn, Cd, Pb and Cu by anodic stripping potentiometry using the oxygen dissolved in the sample as oxidant is reported. The system relies on the use of a PC-compatible computer for instrumental control and data acquisition and processing.


Journal of Automatic Chemistry, Vol. 12, No. 3 (May–June 1990), pp. 108–112

### Automatic system for the determination of metals by anodic stripping potentiometry in non-deaerated samples

**A. Cladera, J. M. Estela and V. Cerdá**
*Department of Chemistry, Faculty of Sciences, University of the Balearic Islands, E07071 Palma de Mallorca, Spain*

*An automatic system for the determination of Zn, Cd, Pb and Cu by anodic stripping potentiometry using the oxygen dissolved in the sample as oxidant is reported. The system relies on the use of a PC-compatible computer for instrumental control and data acquisition and processing.*

### Introduction

The possibility of achieving very low detection limits, and the simplicity and affordability of the instrumentation required [1–4], make anodic stripping potentiometry (ASP) a technique of great interest in the analysis of metal traces in a large variety of samples. However, it has one major disadvantage: as a rule, ASP analyses are time-consuming. In fact, as reported in the literature, the detection limits accomplished by this technique are a function of the pre-electrolysis time used; therefore, the determination of very dilute samples usually involves long pre-electrolysis times.

Jagner [5] proposed the use of the oxygen dissolved in the samples as oxidant in the stripping stage. This offers some advantages over other oxidants: for example the greater simplicity of the analyses resulting from the avoidance of the preliminary deaeration of the samples imposed by such oxidants; the elimination of the potential risk of contamination by the inert gas used in the deaeration; and the greater ease with which analyses can be automated. However, as oxygen is quite a fast oxidant, its use results in a loss of sensitivity compared with other, slower oxidants [6, 7], as it gives rise to shorter plateaux in the potentiometric curves.

The automation of this analytical technique avoids the long time periods otherwise required by offering the possibility of carrying out a large number of analyses in a completely automatic fashion. This paper reports a system devised for this purpose and the results obtained with it. The system is made from straightforward instrumentation, which is controlled by a PC-compatible computer via customized software. The implementation of large numbers of analyses in an automated fashion poses the additional problem of finding a suitable electrode whose surface can be kept stable for long periods. In this respect, a number of electrode generation procedures, based on the formation of a thin layer of mercury over the surface of a glassy carbon electrode, have been tested.

### Instrumentation

The instrumentation used to assemble the automatic system for anodic stripping potentiometric determinations in non-deaerated samples consisted of the following elements: (*a*) a PC-compatible computer; (*b*) a standard potentiostat; (*c*) a commercially available analogue-to-digital converting board (Metrabyte DASH-8, MetraByte Corporation, 440 Myles Standish Boulevard, Taunton, Massachusetts 02780, USA); (*d*) a custom-made optocoupling circuit; (*e*) a Crison (Crison Instruments, SA, Riera Principal 24–26, 08328 Alella, Barcelona, Spain) microBUR-2031 automatic burette furnished with an RS-232C interface; (*f*) a Crison Sampler-2040 sample carousel, also furnished with an RS-232C interface; and (*g*) a set of platinum (counter), calomel (reference) and glassy carbon (working) electrodes. The complete set-up is depicted in figure 1.

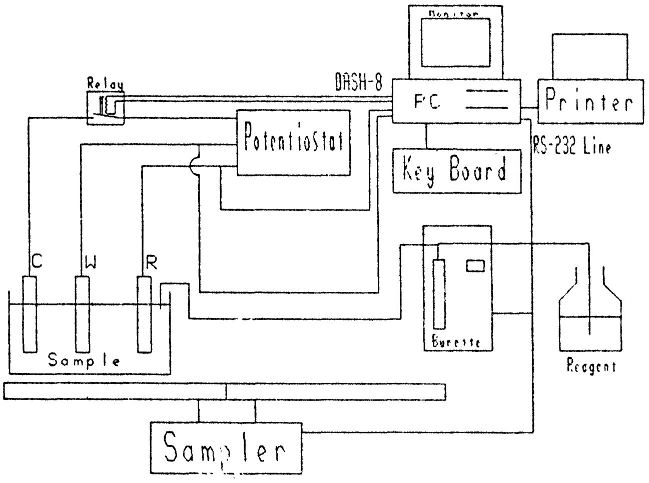

*Figure 1. Block diagram of the automatic system for the ASP determination of metals in non-deaerated samples.*

The computer was used to control all the operations involved, namely the access to the different samples via the carousel, the start and stop of the pre-electrolysis step via the optocoupling circuit, the acquisition of data during the stripping via the A–D board and, finally, the processing of the acquired data. In addition, the automatic burette used, also controlled by the computer, allowed the standard-addition method to be applied automatically by performing successive additions of standard through the burette.

0142–0453/90 $3.00 © 1990 Taylor & Francis Ltd.

*Software*

The software used to run the system allowed the computer to control the instrumentation, the course of the analyses and the processing of the acquired results. It was written in QBASIC and then compiled and linked to the sub-routines governing the operation of the DASH-8 board supplied by the manufacturer. The program consists of two distinct parts which are allocated the experimental realization of the analyses and the processing of the data generated in them, respectively.

*Realization of the analyses*

As stated above, the system includes a carousel which can hold up to 13 samples. The program allows each of the samples to be assayed in turn by applying the required experimental procedure in each instance. This, among other things, requires setting the pre-electrolysis time, the potential range over which data are to be acquired during the stripping, the data acquisition rate and whether or not standard additions are to be performed – and, if so, their features.

*Data processing*

As is widely known, the analytical signal yielded by a given element in ASP is derived from the length of the corresponding plateau in the E–t curve. Two algorithms were assayed which allow such a length to be calculated from the experimental data acquired by the computer. The algorithms were as follows.

*Algorithm A:* the first step in its implementation involves locating the different plateaux from the analysis of the first and second derivative of the E–t curve. A maximum in the first derivative is indicative of an inflection point in the experimental curve and hence the transition from one plateau to the next. Once all plateaux have been located, their lengths are calculated by fitting three straight lines to the experimental curve. Two of such lines include the inflection points which delimit the plateau (one before and another after it), while the third line is fitted to the centre of the plateau. The distance (in seconds) between the two intercepts of the three lines is taken as the analytical signal. Figure 2 illustrates the treatment of a few experimental data; it shows the different fitted lines and the portions of the experimental curve used in each fitting (arrows).

*Algorithm B:* this is a modification of one previously reported by Mortensen *et al.* [8]. It relies on the prior transformation of the experimental data in order to facilitate their processing. Such a transformation involves dividing the working potential range into a number of windows which will depend on the precision required and on the density of the experimental points obtained, and calculating the residence time of the potential of the working electrode in each of the defined windows. Thus, the plot of such a residence time against the potential of each window is a curve in which each element appears as a peak at a characteristic potential. The area under each peak gives a measure of the analytical signal. Figure 3 illustrates the application of this algorithm to the same experimental data used in figure 2. The figure shows the transformed data, as well as the position of the maximum (solid line), the peak amplitude (broken line) and the base-line used to calculate the peak area.

Both algorithms were tested on a large number of experimental data and the results were compared with those obtained by manual processing; consistence was virtually perfect in most instances. Algorithm A appears to be more reliable than B, as it performs calculations on raw data thereby avoiding distortions resulting from their transformation. However, algorithm B was chosen because it is faster than A and is less markedly affected by distortions in the experimental curve arising from excessive noise or other disturbances. Thus, all the results reported in this paper were obtained by applying algorithm B.

### Experimental

*Reagents*

Standard solutions of the elements investigated, viz. Pb(II), Zn(II), Cd(II) and Cu(II), were prepared from their nitrates.

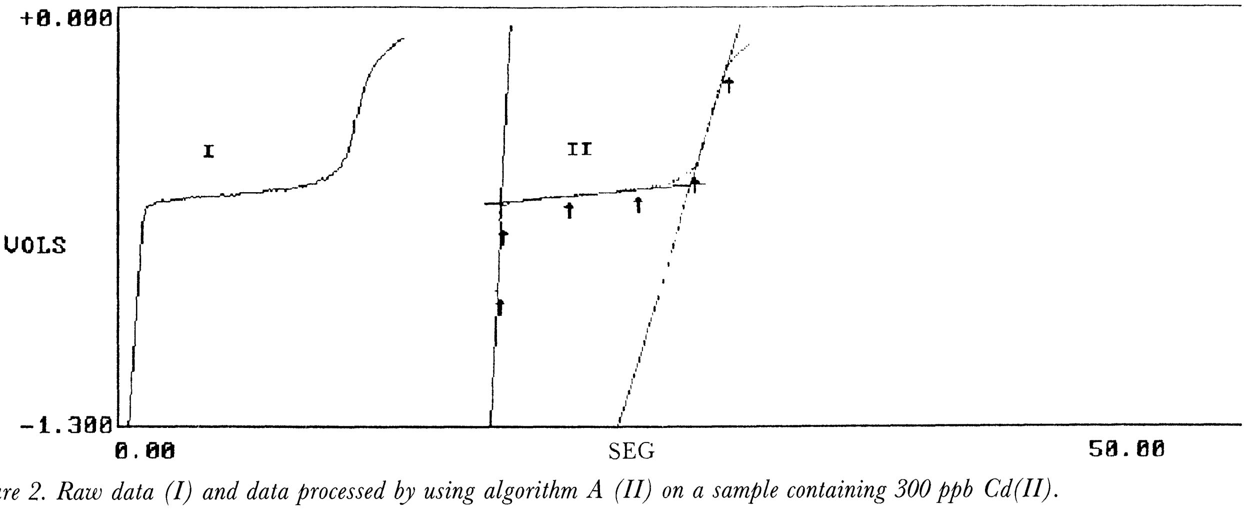

*Figure 2. Raw data (I) and data processed by using algorithm A (II) on a sample containing 300 ppb Cd(II).*

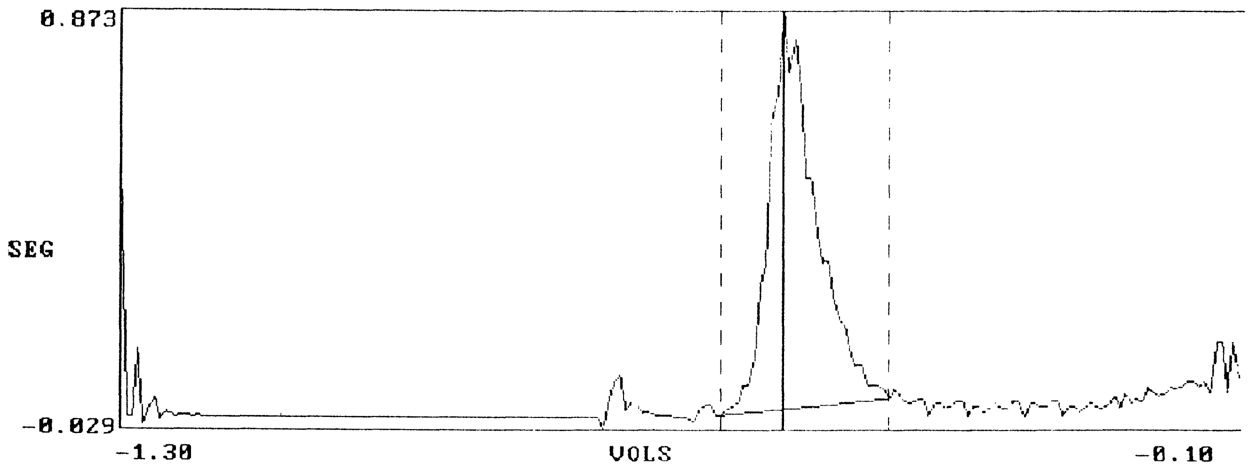

*Figure 3. Data processed by using algorithm B on a sample containing 300 ppb Cd(II).*

A 1000 ppm Hg(II) solution was made from $Hg(NO_3)_2H_2O$.

All reagents used were pro-analysis grade.

*Procedures*

*Generation of the working electrode*

A major problem encountered in applying the ASP technique in an automated fashion lies in developing an experimental procedure allowing the consecutive analysis of large numbers of samples, without the need to regenerate the electrode too frequently while obtaining reliable, reproducible results.

The different procedures for electrode generation reported in the literature usually allow the analysis of only a few samples before the electrode has to be regenerated. This meant that new procedures had to be devised for obtaining longer-lasting electrodes.

After much experimentation, a suitable procedure was developed for each of the elements studied; each procedure allowed a minimum of 13 successive determinations – as many as the carousel capacity allowed for – to be carried out completely automatically without the need to regenerate the electrode. Essentially, the procedures involved implementing a series of plating-stripping cycles aimed at forming and stabilizing a thin layer of metal Hg over the glassy graphite electrode. The basic features of the procedure developed for each element are summarized in table 1.

*Measurement of samples*

Each of the samples had 1 ppm Hg(II) added and was acidified with HCl up to an overall concentration of 0·024 M. The mercury added ensured that the electrode layer remained stable over the analyses. The samples thus prepared were placed on the carousel and assayed, either from a calibration graph or by the standard-addition method. In the former case, the calibration graph was run by using several additions of standards of a solution containing 1 ppm Hg(II) and 0·024 M HCl. The pre-electrolysis time was 30 min in every case, while the pre-electrolysis potential was suited to the element to be determined: −1·3 V for Zn and Cd, −1·0 V for Pb and −0·7 V for Cu.

*Table 1. Procedures for generation of the electrode.*

| | | | | | | | | |
|---|---|---|---|---|---|---|---|---|
| Potential (V) | −0·5 | −0·6 | −0·7 | −0·8 | −0·9 | −0·9 | −1·3 | −1·3 |
| Time (min) | 1 | 1 | 1 | 1 | 1 | 2 | 2 | 30 |

Step 1
(Common to all four elements)
Number of cycles = 1; 80 ppm Hg(II) + 0·1 M HCl

| | Pb | Cd | Cu |
|---|---|---|---|
| Potential | −1·0 | −1·3 | −0·7 |
| Time (min) | 30 | 30 | 30 |

Step 2
(Exclusive to Pb, CD and Cu)
Number of cycles = 5; 2 ppm Hg(II) + 0·03 M $HNO_3$

### Results

For each of the elements studied one calibration graph was run. Then, a series of synthetic samples were assayed by using the calibration graphs previously obtained; finally, samples were determined by the standard-addition method. All the experiments were done automatically and the electrode was only generated prior to starting them.

The results obtained, and the corresponding calibration graphs, are shown in table 2 and in figures 4 and 5, respectively. The results obtained for the different elements assayed are summarized in tables 3–6.

### Conclusions

The anodic stripping potentiometric technique can be applied to non-deaerated samples in an automatic fashion by using straightforward, affordable instrumentation. By increasing the pre-electrolysis time, better

*Table 2. Features of the calibration curves.*

| Element | Equation | *r* | Linear range (ppm) |
|---|---|---|---|
| Zn | $y$ (s) = 0 + 25·30 $x$ (ppm) | 0·997 | 0·02–0·350 |
| Pb | $y$ (s) = 0 + 19·76 $x$ (ppm) | 0·998 | 0·08–0·400 |
| Cd | $y$ (s) = 0 + 28·03 $x$ (ppm) | 0·992 | 0·08–0·400 |
| Cu | $y$ (s) = 0 + 113·3 $x$ (ppm) | 1·000 | 0·08–0·400 |

*Table 3. Results obtained in the determination of Zn.*

| | Calibration graph | | Standard additions | |
|---|---|---|---|---|
| Added (ppm) | Found (ppm) | RSD† (%) | Found (ppm) | RSD (%) |
| 0·04 | 0·040 | 0·2 | 0·039 | — |
| 0·08 | 0·085 | 8·7 | 0·080 | — |
| 0·12 | 0·128 | 4·2 | 0·120 | — |
| 0·16 | 0·168 | 4·1 | 0·155 | — |
| 0·20 | 0·208 | 5·0 | 0·179 | — |
| 0·24 | 0·252 | 5·0 | 0·277 | — |
| 0·28 | 0·304 | 6·2 | 0·264 | — |
| 0·32 | 0·345 | 5·2 | 0·356 | — |
| 0·36 | 0·374 | 9·3 | 0·432 | — |

† Average of the results obtained for four different samples.

*Table 4. Results obtained in the determination of Pb.*

| | Calibration graph | | Standard additions | |
|---|---|---|---|---|
| Added (ppm) | Found (ppm) | RSD† (%) | Found (ppm) | RSD (%) |
| 0·100 | 0·104† | 8·7 | 0·111† | 5·1† |
| 0·200 | 0·201† | 6·8 | 0·179† | 5·8† |
| 0·300 | 0·327† | 2·7 | 0·304† | 10·3† |
| 0·400 | 0·428 | — | 0·422 | — |

† Average of the results obtained for three different samples.

*Table 5. Results obtained in the determination of Cd.*

| | Calibration graph | | Standard additions | |
|---|---|---|---|---|
| Added (ppm) | Found (ppm) | RSD† (%) | Found (ppm) | RSD (%) |
| 0·100 | 0·112† | 9·2 | 0·102† | 5·8† |
| 0·200 | 0·202† | 6·2 | 0·235† | 6·1† |
| 0·300 | 0·319† | 4·9 | 0·350† | 12·4† |
| 0·400 | 0·408 | — | 0·488 | — |

† Average of the results obtained for three different samples.

*Table 6. Results obtained in the determination of Cu.*

| | Calibration graph | | Standard additions | |
|---|---|---|---|---|
| Added (ppm) | Found (ppm) | RSD† (%) | Found (ppm) | RSD (%) |
| 0·080 | 0·086† | 4·1 | 0·080† | 4·8† |
| 0·160 | 0·169† | 7·7 | 0·175† | 6·6† |
| 0·240 | 0·244† | 1·1 | 0·258† | 8·3† |
| 0·320 | 0·291 | — | 0·322 | — |

† Average of the results obtained for three different samples.

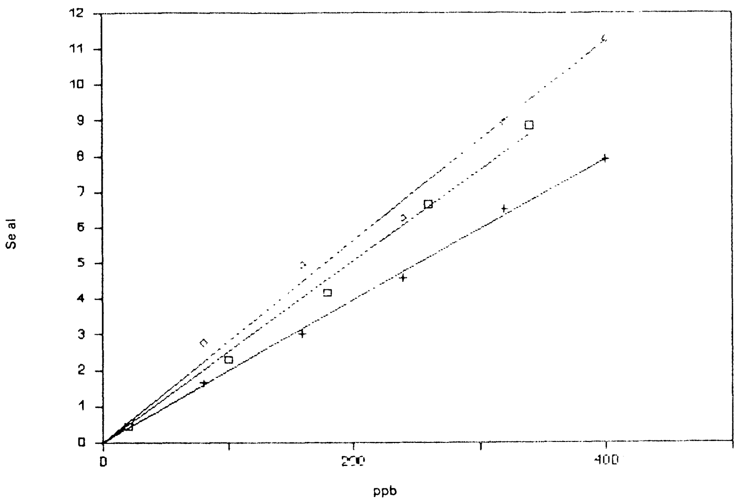

*Figure 4. Calibration graph for Zn (□) Pb (+) and Cd (◇).*

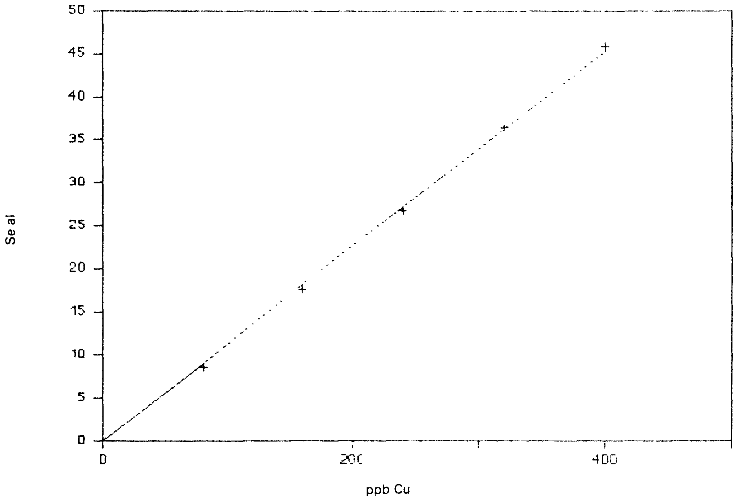

*Figure 5. Calibration graph for Cu.*

determination limits may be achieved (reaching values which are not attained by conventional AAS). On the other hand, potentiometric stripping analysis is a multielemental technique, which decreases the time spent on each element when several electroactive species have to be determined. The elimination of the deaeration step makes it competitive with other considerably more expensive techniques, such as atomic absorption spectrometry, in terms of instrumentation and maintenance costs, especially when the number of samples to be determined is not very high.

The major shortcomings of the ASP technique arise from the reproducibility of the behaviour of the solid electrodes used, which calls for strict control of their use and of the variables affecting their preparation and maintenance.

### Acknowledgement

The authors gratefully acknowledge the financial support of the DGICyT (Spanish Council for Research in Science and Technology) (PA 86-0033).

### References

1. Jagner, D. and A. Graneli, A., *Analytica Chimica Acta*, **83** (1976), 19.

2. Jagner, D., *Analytical Chemistry*, **50** (1978), 1924.

3. Chi Chau, T., Yu Li, D. and Liang Wu, Y., *Talanta*, **29** (1982), 1083.

4. Jagner, D., *Analyst*, **107** (1982), 593.

5. Jagner, D., *Analytical Chemistry*, **51** (1979), 343.

6. Jagner, D. and Arén, K., *Aanlytica Chimica Acta*, **107** (1979), 29.

7. Jagner, D. and Westerlund, S., *Analytica Chimica Acta*, **117** (1980), 159.

8. Mortensen, J., Ouziel, E., Skov, H. J. and Kryger, L., *Analytica Chimica Acta*, **112** (1979), 297.

